# An evolutionary approach to a combined mixed integer programming model of seaside operations as arise in container ports

**DOI:** 10.1007/s10479-017-2539-7

**Published:** 2017-05-22

**Authors:** Abdellah Salhi, Ghazwan Alsoufi, Xinan Yang

**Affiliations:** 1 Department of Mathematical Sciences, University of Essex, Colchester, UK; 2 College of Computer Sciences and Mathematics, University of Mosul, Mosul, Iraq

**Keywords:** Container terminals, Berth allocation, Quay crane assignment, Quay crane scheduling, Mixed integer programming, Genetic algorithm

## Abstract

This paper puts forward an integrated optimisation model that combines three distinct problems, namely berth allocation, quay crane assignment, and quay crane scheduling that arise in container ports. Each one of these problems is difficult to solve in its own right. However, solving them individually leads almost surely to sub-optimal solutions. Hence, it is desirable to solve them in a combined form. The model is of the mixed-integer programming type with the objective being to minimize the tardiness of vessels and reduce the cost of berthing. Experimental results show that relatively small instances of the proposed model can be solved exactly using CPLEX. Large scale instances, however, can only be solved in reasonable times using heuristics. Here, an implementation of the genetic algorithm is considered. The effectiveness of this implementation is tested against CPLEX on small to medium size instances of the combined model. Larger size instances were also solved with the genetic algorithm, showing that this approach is capable of finding the optimal or near optimal solutions in realistic times.

## Introduction

Container terminals are important assets in many modern economies. They are expensive to build, and difficult to operate. Some of the main operations, also known as seaside operations, faced daily by decision makers in these terminals are

1. Berth allocation,
2. Quay crane assignment,
3. Quay crane scheduling.

Landside operations include yard planning, yard crane assignment, container storage planning and many others. In this paper, only the seaside problems listed above are considered. Note that each one of them is a complex optimization problem in its own right (Bierwirth and Meisel 2010, 2015; Steenken et al. 2004). However, solving them individually, independently of each other, leads almost certainly to overall suboptimal solutions. It is therefore desirable to investigate the triple integrated problem as a single model. This is the object of this paper.

Operations at container terminals are usually sequenced as follows. First, a vessel arriving at the port is allocated a berth and that consists of a berthing time and a berthing position. This is known as the berth allocation Problem or BAP. The objective of this problem is to minimize the total vessel turnaround time, which is the sum of the handling and waiting times of each vessel, i.e. the total time of unloading and loading containers from/onto a vessel, depending on the distance between its berthing position and the desired berthing one. The desired berthing position is the position which has the minimum distance from the pre-allocated yard storage in the port, where the containers will be stored until they are transferred to intermediary or final destinations. The decision makers in the container terminal consider jointly the best berthing time as well as the best berthing position, so as to minimize the turnover time and the movement cost simultaneously. Based on the berthing plan, the second operation, known as the Quay Crane Assignment Problem or QCAP, tries to determine the optimum number of quay cranes to allocate to every vessel so that the throughput of cranes is maximized or, equivalently, their idle time is minimized. Therefore, the handling times of vessels are expected to be minimized so as to meet their expected finishing times (due times). The last operation is the quay crane scheduling problem or QCSP. It strives to find the optimum order in which to carry out the tasks on vessels in order to minimize their processing time. This depends on how many quay cranes are available to use on every vessel (the output of QCAP), and the berthing time and position of the vessel (the output of BAP).

It is often assumed in the relevant literature, as we shall see later, that a quay crane cannot move to another vessel before the processing of the one it is working on has finished. In our model BACASP, which integrates berth allocation, quay crane assignment and quay crane scheduling, quay cranes can move from vessel to vessel as need may be irrelevant of whether the vessel they are leaving is still under processing or not, provided it is profitable to do so, and no interference between quay cranes is created. BACASP is solved with an adapted variant of the genetic algorithm as well as Branch-and-Cut as implemented in CPLEX. Comparative results are drawn.

This paper is organised as follows. Section 2 is a literature review. The problem description is given in Sect. 3. BACASP, the full mathematical model is proposed in Sect. 4. The GA and its implementation to handle BACASP is presented in Sect. 5. Section 6 records computational results. Section 7 is the conclusion and suggestions for future work.

## Problems under consideration: a review

As said earlier, berth allocation, assigning and scheduling quay cranes are at the heart of container port operations. Here, we review the relevant literature on these problems and related ones.

### The berth allocation problem

BAP with continuous wharf is the normal setting in most modern container ports as it offers more flexibility. Our study, therefore, assumes a continuous wharf setting too. Under similar problem settings, Li et al. (1998) formulated the problem in the “multiple-job-on-one-processor” model, where multiple jobs refer to several vessels and the one processor refers to a single berth. A small vessel moored in a berth may share it with other vessels if their total length does not exceed the length of the wharf. Lim (1998) formulated BAP and considered that the berthing time is equal to the arrival time of each vessel and by solving his model, a berthing position is found which minimizes the maximum quay length required to serve vessels in accordance with the schedule. Moon (2000) proposed a mixed integer linear programming model whose objective is to minimize the tardiness of vessels. A Lagrangian relaxation representation of the integer programming model is then solved by a sub-gradient optimization algorithm. Goh and Lim (2000) discussed the methods of modelling BAP and proposed several approaches to solve it such as Randomized Local Search (RLS), GA and Tabu Search (TS). Kim and Moon (2003) formulated BAP as a mixed integer program and applied a simulated annealing algorithm to find near optimal solutions. Guan and Cheung (2004) developed two inter-related BAP mathematical models the objective functions of which are to minimize the total weighted finishing time of vessels. A tree search procedure is used to solve the first model. This provides a good lower bound which speeds up the tree search procedure applied to the second one.

Imai et al. (2005) addressed BAP in a multi-user container terminal with a continues wharf and introduced a nonlinear programming model to represent it. They used a heuristic approach to solve it. They have discussed the preference for a flexible berth layout which has become very important especially in busy hub ports where ships of various sizes dock. Wang and Lim (2007) transformed BAP into a multiple stage decision making problem and a new multi-stage search method, namely the Stochastic Beam Search algorithm, was used to solve it. Lee and Chen (2009) proposed a neighborhood-search based heuristic to determine the berthing time and space for each incoming vessel to the continuous berth stretch. In their method, the First-Come-First-Served rule, a clearance distance between vessels and the possibility of vessel shifting, were considered. Lee et al. (2010) studied the continuous and dynamic BAP in order to minimize the total weighted flow time. The authors follow the mathematical model of Guan and Cheung (2004). Two versions of the Greedy Randomized Adaptive Search Procedures (GRASP) heuristic were developed to find near optimal solutions. Ganji et al. (2010) proposed a GA for large scale BAPs which we adapted ourselves in this paper to find optimal or near optimal solutions. Cheong et al. (2010) solved BAP by using multi-objective optimization in order to minimize concurrently the three objectives of makespan, waiting time, and degree of deviation from a predetermined priority schedule. These three objectives represent the interests of both port and ship operators. Unlike most existing approaches in single-objective optimization, multi-objective evolutionary algorithm (MOEA) incorporates the concept of Pareto optimality which is used when solving the multi-objective BAP. Oliveira et al. (2012) proposed a heuristic to solve a continuous case of BAP. This heuristic is based on the application of the clustering search method with simulated annealing. Xu et al. (2012) studied robust berth allocation. Their approach to mitigating uncertainty consists in inserting time buffers between the vessels occupying the same berthing location. Using the total departure delay of vessels as the service measure and the length of the time buffer as the robustness measure, the authors formulated a robust BAP or RBAP with the aim of balancing the service level and the plan robustness. They solved their model with a hybrid meta-heuristic that integrates simulated annealing and Branch-and-Bound. It is important to note that they consider only tardiness in the objective rather than the preferred berthing location. This, we believe, may potentially affect adversely yard management. Alsoufi et al. (2016) also proposed a mathematical model for robust berth allocation. The problem is first formulated as a mixed-integer programming model whose main objective is to minimize the total tardiness of vessel departure time and reduce the cost of berthing. A hybrid meta-heuristic based on GA and Branch-and-Cut algorithms (GA+B&C), is implemented to find optimal or near optimal solutions to large scale instances of this problem.

### The quay crane scheduling problem

The solution of QCSP is the optimal sequence of moves the quay cranes must perform when unloading/loading containers from/onto vessels in order to minimize the handling time of these vessels.

Kim and Park (2004) studied QCSP and formulated it as a mixed integer programme. They used Branch-and-Bound (B&B) in conjunction with the Greedy Randomized Adaptive Search Procedure (GRASP) (Feo and Resende 1995), to overcome the difficulties of B&B on its own. Moccia et al. (2006), formulated QCSP as a vehicle routing problem with additional constraints like the precedence relationships between tasks. CPLEX was used to solve small scale instances and for larger ones they developed a variant of the B&C algorithm which incorporates a number of valid inequalities and exploits precedence constraints. Sammarra et al. (2007) proposed a Tabu Search heuristic to minimize the completion time for unloading and loading containers. They considered precedence as well as non-simultaneity between tasks. They observed that QCSP can be decomposed into a routing problem and a scheduling problem. Lee et al. (2008) presented a mixed integer programming model of QCSP and proved that it is NP-complete; they used a GA to solve problem instances to near optimality. Bierwirth and Meisel (2009) noticed the shortcomings of earlier models of QCSP, and in particular with respect to the quay crane interference avoiding constraints which did not do the job properly. They revised one such model due to Sammarra et al. (2007) to take care more appropriately of interference between quay cranes. They also proposed a Unidirectional Schedule (UDS) heuristic for when the quay cranes do not change moving direction from their initial position and have identical directions of movement both in upper and lower bays. Chung and Choy (2012) proposed a variant of GA to solve Kim and Park’s model of QCSP (Kim and Park 2004). Their results compared well with those obtained by most well-established algorithms. Kaveshgar et al. (2012) introduced an efficient GA for QCSP. Their algorithm improved the efficiency of GA search by using an initial solution based on the S-LOAD rule and by reducing the number of genes in the chromosomes to reduce search time. Nguyen et al. (2013) suggested two representations of QCSP one for GA and the other for Genetic Programming (GP) (Koza 1992). GA uses permutation to decide the priority of tasks, whereas GP relies on a priority function to calculate the priority of tasks. Unsal and Ceyda (2013) proposed a constraint programming approach for solving QCSP. In Guan et al. (2013) the crane scheduling problem for a moored vessel is studied. Both exact and heuristic solution approaches for the problem have been suggested. Small size instances have been formulated using a time-space network flows with non-crossing constraints. Optimal solutions were found for these instances using exact solution approaches. For large size instances, two heuristics have been used. The authors report promising results based on their limited experimentation.

### The combined aerth allocation and quay crane assignment problem (BACAP)

QCAP is the problem of finding the optimum number of quay cranes that should be assigned to every vessel that docks at a container terminal. This problem can be seen as trivial since knowing the workload of a vessel and the work rate of a quay crane should allow to estimate the number of quay cranes required for that vessel. However, if quay cranes of different work rates are used and are allowed to move from one ship to another while work on the ships is ongoing, then their assignment is no longer so simple. BACAP is the problem of allocating berthing times and berthing positions to vessels and, at the same time, determining the optimum number of quay cranes to service them.

This problem has also been well studied. Legato et al. (2008), addressed QCAP with predetermined berth position and time following the solution of BAP. They assumed that quay cranes could not move between vessels before all tasks are performed and the processing of the concerned vessels is finished. A mathematical model was presented to determine the optimum number of quay cranes for each vessel that is ready for processing. Meisel and Bierwirth (2009) combined BAP and QCAP into BACAP. The proposed problem is formulated taking into account some of the real issues faced by the decision maker at the port. In addition to the mathematical model, they also suggested two meta-heuristic approaches for the problem: the Squeaky Wheel Optimization (SWO), and Tabu Search (TS). Cheong et al. (2010) considered the multi-objective optimization aspect of BACAP; indeed, it involves simultaneous optimization of two highly-coupled container terminal operations. Optimization results show that the multi-objective approach offers the port manager flexibility in selecting a desirable solution for implementation. Yang et al. (2012) suggested to deal with BACAP by simultaneously solving BAP and QCAP. They formulated a mathematical model which integrates the BAP constraints of Guan and Cheung (2004) and the QCAP constraints of Legato et al. (2008). The objective function for this model is the combination of the objective functions of the BAP and QCAP models. An evolutionary algorithm was developed to find the solution to this coarse combined problem.

### The combined quay crane assignment and quay crane scheduling problem (QCASP)

The decision maker in QCASP focuses on determining the number of quay cranes for each vessel and finding the best sequence in which tasks will be performed by these quay cranes. There is obviously a strong relationship between the two goals.

Daganzo (1989) addressed quay crane scheduling for multiple vessel arrivals. They proposed both an exact and an approximate solution approach with the objective being to minimise the tardiness of all vessels. Peterkofsky and Daganzo (1990) developed a B&B algorithm to solve QCSP. Interference between quay cranes has not been considered in both papers. Tavakkoli-Moghaddam et al. (2009) studied QCASP. They formulated a mixed integer programme to determine the optimal number of quay cranes for every vessel that arrives at the terminal and at the same time the optimal sequence in which the tasks should be carried out. An evolutionary approach (GA) is suggested to solve large scale instances of this problem. Unsal and Ceyda (2013) extended their constraint programming model for QCSP to deal with QCASP. Diabat and Theodorou (2014) and Fu et al. (2014) also proposed a combined model for QCASP and implemented a variant of GA for large scale instances. Their model allows the movement of quay cranes between vessels while the processing of vessels is ongoing. This is achieved by discretizing the time horizon and using a time index. The interference avoiding constraint is represented simply by the position of quay cranes at each short time interval. This increases the number of variables in the model and potentially makes the problem difficult to solve (curse of dimensionality). Note that time discretization makes the model less accurate. The omission of quay crane travelling time between bays/vessels makes the solution of Fu et al. (2014) impractical, especially when the frequency of quay crane movement is high. Precedence and simultaneity constraints are also not considered. Note that the results reported in Fu et al. (2014) in Table 7 for instance do not make sense. The GAMS results which presumably are exact, cannot possibly be worse that those returned by GA for most of the problem instances considered.

### Combining berth allocation, quay crane assignment, and quay crane scheduling problems

Ak and Erera (2006) present a mixed integer linear programming model of the seaside operations. A two phase tabu search heuristic is implemented for instances of the model involving low numbers of vessels containing between 3 and 4 holds. The travelling time of quay cranes is not considered and there is no penalty if a vessel is moored at an unsuitable position. The simultaneity and precedence constraints are not involved in the model. Criteria such as the initial position, the travelling time cost and ready time of quay cranes are ignored.

Liu et al. (2006) studied the seaside operations and use mixed integer linear programming to minimize the maximum relative tardiness of vessel departures. A good idea presented by Liu was that instead of assuming a relationship function to exist between the processing time of a vessel and the number of quay cranes are assigned to it, one should introduce a series of parameters $p_{vj}$ each of which referring to the handling time of vessel *j* when *v* quay cranes are assigned to it. However, the proposed integration model needs further improvement since in the Berth-level model, and the berthing times are revised, whereas the berthing positions are taken from the tentative berth plan.

Meisel and Bierwirth (2013) proposed a three-phase integration framework using preprocessing and feedback mechanisms. Phase I estimates productivity rates of quay cranes from the vessel stowage plan. In phase II, using Meisel’s model (Meisel and Bierwirth 2009) the berth allocation problem and quay crane assignment problem are solved depending on the productivity rates of quay cranes. Phase III schedules the tasks of each vessel. To adjust the solution for BAP, QCAP, and QCSP a feedback loop is needed. Based on this framework, seaside planning problems for 40 vessels are solvable in reasonable computation times.

Rodriguez-Molins et al. (2014), the focus is on BAP and QCAP problems. However, given that the overall problem is a transshipment one, they also have to deal with laoding/unloading. It is therefore of the combined type as discussed in this section. The QC scheduling aspect is handled through a management of vessel holds. They dealt with the overall problem by applying the Greedy Randomized Adaptive Search Procedures (GRASP) heuristic which solves the integrated problem in reasonable times.

Turkogullari et al. (2016) integrated the seaside operations into a single MILP model and proposed a decomposition algorithm for it. The authors achieved the model and its solution on the back of a list of eight assumptions amongst which discretizing the time horizon and making use of a time index in the mathematical model, are perhaps the most important. The anti-interference constraint is represented simply by the position of quay cranes at every small time interval. This increases the number of variables including the integer ones, which potentially, increases the difficulty of the model. Also they assumed that all the quay cranes are available when assigning quay cranes to vessels. They computed the processing time of vessels by dividing the number of containers upon each vessel over all the assigned quay cranes to this vessel. The quay cranes starting point is ignored in their model. The precedence and simultaneity constraints were also not considered. They assumed that all tasks (containers) are located in the same bay. Therefore, $\lambda$, the interference parameter between quay cranes and their travelling time are also ignored. Some vessels have large deviations from their desired berthing positions. Moreover, the advocated solution approach requires many parameters some of which are not easy to estimate and may affect the solution if good values are not used. Examples of such parameters are the minimum and maximum number of quay cranes. Note that the discretization makes the model less accurate than the one we are presenting in this work.

## Problem description

The berth allocation problem attempts to find the best time for berthing and the best position for mooring the vessels that arrive at the container terminal. After berth allocation, the number of quay cranes to assign to every vessel is decided; too low, processing the vessel will take too long; the tardiness of vessels has a knock on effect on the operations of the container terminal as it may delay the processing of other vessels. If, on the other hand, the assigned number of QC’s to vessels is too high, then other vessels will not have enough and therefore their processing will take longer than necessary. The problem which follows immediately is that of finding the sequence in which tasks on each vessel are carried out. Choosing the best sequence to perform these tasks helps minimize the finishing time of vessels.

**Fig. 1:**
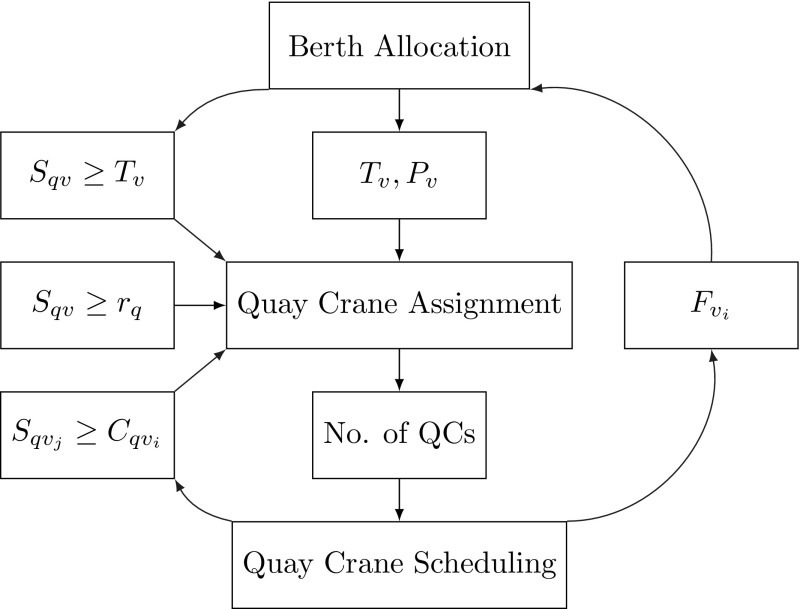
Relationships among the three operations

All these problems have been solved either individually or integrated pairwise. The results obtained might be optimal, however, individual optimal solutions do not guarantee overall optimality. Figure 1 shows the relationship between the different operations.

Note that the solution of BAP returns the berthing time $T_v$ and the berthing position $P_v$ of vessel *v*. These outputs are used as input to the solution procedure of QCAP to determine the appropriate number of quay cranes for vessel *v*, e.g. the starting time $S_{qv}$ of the *q*th quay crane on vessel *v* should be no later than the berthing time $T_v$, and the ready time of the quay crane; the processing time of a single task depends on the berthing location of the vessel, etc. After determining the number of quay cranes for each vessel, the quay cranes have to be scheduled through solving QCSP to choose the best order in which to perform the tasks. This affects the processing time of each vessel.

## Mathematical formulation

### Assumptions

Consider a container terminal with a continuous and fixed length wharf where vessels can moor at at their preferred positions whenever possible. Now assume that

1. Each vessel is divided longitudinally into bays; all bays have the same length. Thus, the length of vessels is in numbers of bays.
2. The safety distance between each pair of adjacent quay cranes depends on the width of a bay.
3. Once a quay crane starts processing a task, it can leave only after it has finished the task.
4. Any vessel can be processed in any space of the wharf depending on the arrival time and the available terminal.
5. Quay cranes are on the same rail and thus cannot cross over each other.
6. Some tasks must be performed before others and tasks that can be performed simultaneously.

### Indices

*Q*: Number of quay cranes $(q,q_i,q_j=1,2, \ldots , Q)$.
*V*: Number of vessels $(v,v_i,v_j=1,2, \ldots ,V)$.
$$B_v $$
Number of tasks on vessel v $(b,b_i,b_j=1,2, \ldots ,B_v)$.

### Parameters

$$p_{b}^v$$
Time required to perform task *b* on vessel *v*.
$$l_{b}^v$$
Location of task *b* on vessel *v* expressed by the ship bay number on vessel *v*.
$$r_q$$
Earliest available time of the *q*th quay crane.
$$l_0^q$$
Initial location of quay crane *q* which is relative to the ship-bay number.
$$l_T^q$$
Final location of quay crane *q* which is relative to the ship-bay number.
$$t_{b_ib_j}^{qv}$$
Travel time of the *q*th quay crane from the location $$l_{b_i}^v$$ of task $${b_i}$$ to the location $$l_{b_j}^v$$ of task $${b_j} $$. $$t_{b_0b_j}^{qv}$$ represents the travel time from the initial position $$l_q^v$$ of the *q*th quay crane to the location $$l_{b_j}^v$$ of the task $${b_j} $$ on vessel *v*. In addition, $$t_{b_jb_{{B_v}+1}}^{qv}$$ represents the travel time from location $$l_{b_j}^v$$ of task $${b_j} $$ to the final destination $$l_q^v$$ of *q*th quay crane on vessel *v*. Where tasks $$b_0$$ and $$b_{{B_v}+1}$$ are considered as the dummy initial and final states of each quay crane, respec- tively.
$$a_v$$
Estimated arrival time for vessel *v*.
$$d_v$$
Requested departure time for vessel *v*.
$$\hat{P}_v$$
Preferred berth position of vessel *v*. It is determined by the position of yard storage areas allocated to vessel *v*. $\hat{P}_v$ means that the containers on ship *v* are at the shortest distance from their allocated yard storage.
$$U_v$$
Distance cost due to vessel *v* not being moored at $\hat{P}_v$, its preferred berthing position.
$$L_v$$
Length of the vessel *v*.
*W*: Length of the wharf.
$$W_v$$
Tardiness cost of vessel *v* per time unit.
$$R_v$$
Earliness income of vessel *v* per time unit.
$$\Psi $$
Set of pairs of tasks that cannot be performed simultaneously; when tasks $$b_i $$ and $$b_j $$ cannot be performed simultaneously, then $$(b_i,b_j)\in \Psi $$.
$$\Phi $$
Set of ordered pairs of tasks for which there is a precedence relationship; when task $$b_i $$ must precede task $$b_j$$, then $$(b_i,b_j) \in \Phi $$.
*M*: Arbitrarily large positive number.

### Binary decision variables

$$\begin{aligned} X_{b_ib_j}^{qv}= & {} {\left\{ \begin{array}{ll} 1 &{} \text {if the }q \mathrm{th}\text { quay crane performs task }b_j\text { immediately after performing task }b_i\text { on vessel }v. \\ 0 &{} \text {otherwise} \end{array}\right. } \end{aligned}$$

Tasks $b_0$ and $b_{{B_v}+1}$ are considered as the dummy initial and final states of each quay crane, respectively. Thus, when task $b_j$ is the first task of the *q*th quay crane, then $X_{b_0b_j}^{qv}=1$. In addition, when task $b_j$ is the last task of the *q*th quay crane, then $X_{b_jb_{{B_v+1}}}^{qv}=1$.

$$\begin{aligned} Z_{b_ib_j}^v= & {} {\left\{ \begin{array}{ll} 1 &{} \text {if task }b_j\text { starts later than the end of task }b_i\text { on vessel }v. \\ 0 &{} \text {otherwise.} \end{array}\right. }\\ Y_{v_i v_j}^q= & {} {\left\{ \begin{array}{ll} 1 &{} \text {if the }q \mathrm{th}\text { quay crane is assigned to vessel }v_j\text { right after finishing its tasks on vessel }v_i. \\ 0 &{} \text {otherwise.} \end{array}\right. }\\ \delta _{v_i v_j}= & {} {\left\{ \begin{array}{ll} 1 &{} \text {if the processing of vessel }v_j\text { starts later than the end of vessel }v_i. \\ 0 &{} \text {otherwise.} \end{array}\right. }\\ \sigma _{v_i v_j}= & {} {\left\{ \begin{array}{ll} 1 &{} \text {if the vessel }v_i\text { is located below the vessel }v_j\text { in the berth (wharf).} \\ 0 &{} \text {otherwise.} \end{array}\right. }\\ \beta _{b_ib_j}^{v_i v_j}= & {} {\left\{ \begin{array}{ll} 1 &{} \text {if the task }b_j\text { on vessel }v_j\text { starts later than the finishing of task }b_i \text { on vessel }v_i. \\ 0 &{} \text {otherwise.} \end{array}\right. }\\ \alpha _{b_ib_j}^{v_i v_j}= & {} {\left\{ \begin{array}{ll} 1 &{} \text {if the task }b_i\text { on vessel }v_i\text { is located below the task }b_j\text { on vessel }v_j. \\ 0 &{} \text {otherwise.} \end{array}\right. } \end{aligned}$$

### Continuous decision variables

$$T_v$$
Berthing time of vessel *v*.
$$P_v$$
Berthing position of vessel *v*.
$$A_v$$
Tardiness of vessel *v*.
$$E_v$$
Earliness of vessel *v*.
$$C_{q v}$$
Completion time of *q*th quay crane on vessel *v*.
$$F_v$$
Finishing time of vessel *v*.
$$D_{b_i}^v$$
Completion time of task $b_i$ on vessel *v*.
$$S_{q v}$$
Starting time of *q*th quay crane on vessel *v*.

### The mathematical model

1 $$\begin{aligned}&\min Z = \sum _{v=1}^V W_vA_v-\sum _{v=1}^V R_vE_v+\sum _{v=1}^V U_v|P_v-\hat{P}_v|\\&\text{ s.t }&\nonumber \end{aligned}$$

2 $$\begin{aligned}&d_v-F_v = E_v-A_v&\forall v \end{aligned}$$

3 $$\begin{aligned}&F_{v_i}\le T_{v_j}+M(1-\delta _{v_i v_j})&\forall v_i,v_j;v_i\ne v_j \end{aligned}$$

4 $$\begin{aligned}&P_{v_i}+L_{v_i}\le P_{v_j}+M(1-\sigma _{v_i v_j})&\forall v_i,v_j;v_i\ne v_j \end{aligned}$$

5 $$\begin{aligned}&\sigma _{v_i v_j}+\sigma _{v_j v_i}+\delta _{v_i v_j}+\delta _{v_j v_i}\ge 1&\forall v_i,v_j;v_i\ne v_j \end{aligned}$$

6 $$\begin{aligned}&a_v \le T_v&\forall v \end{aligned}$$

7 $$\begin{aligned}&P_v+L_v\le W&\forall v \end{aligned}$$

8 $$\begin{aligned}&\sum _{v_j=1}^V Y_{v_0 v_j}^q= 1&\forall q \end{aligned}$$

9 $$\begin{aligned}&\sum _{v_i=1}^V Y_{v_i (V+1)}^q= 1&\forall q \end{aligned}$$

10 $$\begin{aligned}&\sum _{v_j=1}^{V+1} Y_{v v_j}^q-\sum _{v_j=0}^V Y_{v_j v}^q = 0&\forall v,q \end{aligned}$$

11 $$\begin{aligned}&\sum _{v_i=0}^V \sum _{q=1}^Q Y_{v_i v}^q\ge 1&\forall v \end{aligned}$$

12 $$\begin{aligned}&S_{qv}\ge r_q -M(1-Y_{v_0 v}^q)&\forall v,q \end{aligned}$$

13 $$\begin{aligned}&S_{qv}\ge T_v-M(1-\sum _{v_j=1}^{V+1} Y_{v v_j}^q)&\forall v,q \end{aligned}$$

14 $$\begin{aligned}&S_{qv_j}\ge C_{qv_i} -M(1- Y_{v_i v_j}^q)&\forall v_i,v_j;v_i\ne v_j;q \end{aligned}$$

15 $$\begin{aligned}&\sum _{b_j=1}^{B_v} X_{b_0b_j}^{qv} = \sum _{v_i=0}^V Y_{v_i v}^q&\forall v,q \end{aligned}$$

16 $$\begin{aligned}&\sum _{b_j=1}^{B_v} X_{b_jb_{{B_v}+1}}^{qv} = \sum _{v_i=0}^V Y_{v_i v}^q&\forall v,q \end{aligned}$$

17 $$\begin{aligned}&\sum _{b_j=1}^{{B_v}+1} X_{b b_j}^{qv}-\sum _{b_j=0}^{B_v} X_{b_jb}^{qv}=0&\forall b,v,q \end{aligned}$$

18 $$\begin{aligned}&\sum _{q=1}^Q \sum _{b_i=0}^{B_v} X_{b_ib}^{qv}= 1&\forall b, v \end{aligned}$$

19 $$\begin{aligned}&\sum _{b_i=0}^{B_v} \sum _{b_j=1}^{{B_v}+1} X_{b_ib_j}^{qv}\le M\sum _{v_i=0}^V Y_{v_i v}^q&\forall v,q \end{aligned}$$

20 $$\begin{aligned}&D_{b_i}^v+t_{b_ib_j}^{qv}+p_{b_j}^v-D_{b_j}^v \le M(1-X_{b_ib_j}^{qv})&\forall b_i,b_j;b_i\ne b_j,v,q \end{aligned}$$

21 $$\begin{aligned}&S_{qv}+t_{b_0b_j}^{qv}+p_{b_j}^v-D_{b_j}^v\le M(1-X_{b_0b_j}^{qv})&\forall b_j,v,q \end{aligned}$$

22 $$\begin{aligned}&D_{b_j}^v-C_{q v}\le M(1-X_{b_jb_{{B_v}+1}}^{qv})&\forall b_j,v,q \end{aligned}$$

23 $$\begin{aligned}&C_{q v}-F_v\le M(1-\sum _{v_j=1}^{V+1} Y_{v v_j}^q)&\forall v,q \end{aligned}$$

24 $$\begin{aligned}&D_{b_i}^v+p_{b_j}^v\le D_{b_j}^v&\forall (b_i,b_j)\in \Phi _v;b_j\ne b_i; \forall v \end{aligned}$$

25 $$\begin{aligned}&D_{b_i}^v-D_{b_j}^v+p_{b_j}^v \le M(1-Z_{b_ib_j}^{v})&\forall b_i,b_j;b_i\ne b_j;\forall v \end{aligned}$$

26 $$\begin{aligned}&Z_{b_ib_j}^{v}+Z_{b_jb_i}^{v}=1&\forall (b_i,b_j)\in \Psi _v;b_j\ne b_i; \forall v \end{aligned}$$

27 $$\begin{aligned}&\sum _{\theta =0}^Q\sum _{\kappa =0}^{B_v} X_{\kappa b_j}^{\theta v}-\sum _{\theta =0}^Q\sum _{\kappa =0}^{B_v} X_{\kappa b_i}^{\theta v}\le M(Z_{b_ib_j}^{v}+Z_{b_jb_i}^{v})&\forall b_i,b_j;b_i\ne b_j;l_{b_i}<l_{b_j};\forall v,q \end{aligned}$$

28 $$\begin{aligned}&P_{v_i}+l_{b_i}^v\le P_{v_j}+l_{b_j}^v+M(1-\alpha _{b_ib_j}^{v_i v_j})&\forall b_i,b_j, v_i,v_j;v_j\ne v_i \end{aligned}$$

29 $$\begin{aligned}&D_{b_i}^{v_i}-D_{b_j}^{v_j}+p_{b_j}^{v_j}\le M(1-\beta _{b_ib_j}^{v_i v_j})&\forall b_i,b_j, v_i,v_j;v_j\ne v_i \end{aligned}$$

30 $$\begin{aligned}&\beta _{b_ib_j}^{v_i v_j}+\beta _{b_jb_i}^{v_j v_i}+\alpha _{b_jb_i}^{v_j v_i}\ge \sum _{\kappa =0}^{Bv} X_{\kappa b_i}^{{q_i} {v_i}}+\sum _{\kappa =0}^{Bv} X_{\kappa b_j}^{{q_j}{v_j}}-1&\forall b_i,b_j, v_i,v_j,q_i,q_j;v_j\ne v_i;q_i<q_j \end{aligned}$$

31 $$\begin{aligned}&X_{b_ib_j}^{qv},Z_{b_ib_j}^{v},Y_{v_i v_j}^q,\delta _{v_i v_j},\sigma _{v_i v_j},\alpha _{b_ib_j}^{v_i v_j},\beta _{b_ib_j}^{v_i v_j} \in \{0,1\} \end{aligned}$$

32 $$\begin{aligned}&C_{q v},F_v,D_{b_j}^v,P_v,T_v\ge 0 \end{aligned}$$

**Fig. 2:**
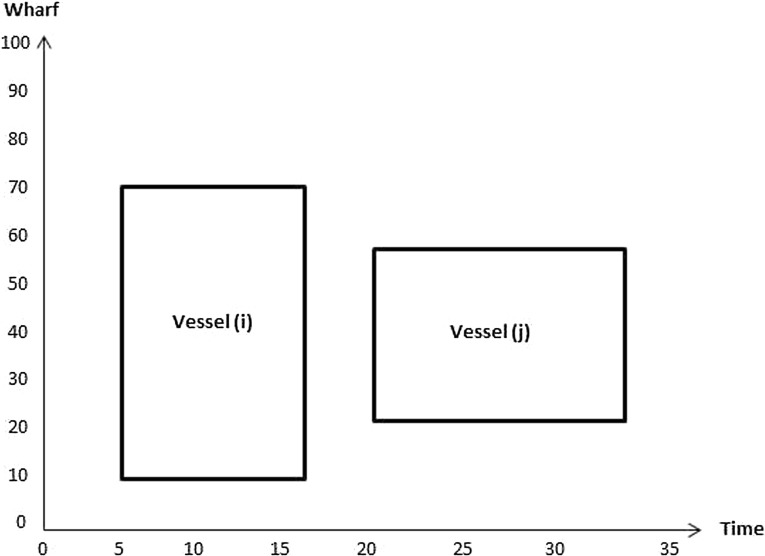
Illustration of no overlap in time between vessels *i* and *j*

**Fig. 3:**
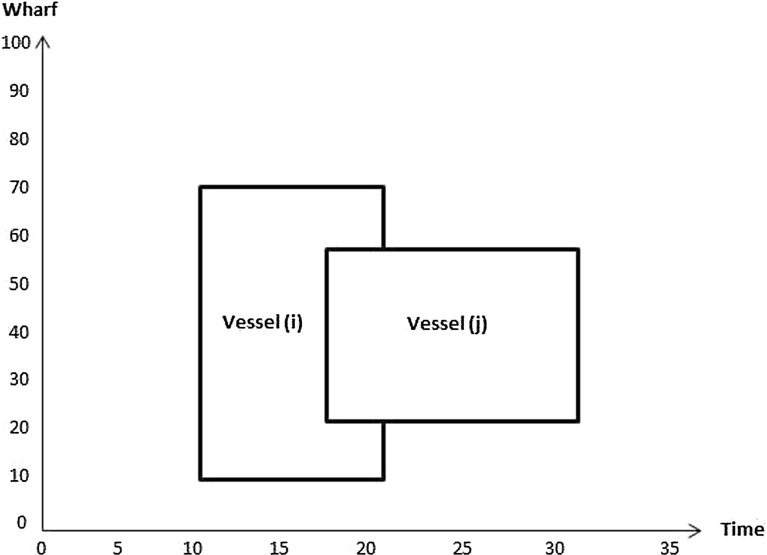
Illustration of overlap in time between vessels *i* and *j*

In the objective function (1), the first term $\sum _{v=1}^V W_vA_v$ represents the tardiness cost if the departure time of the vessel is later than its due time. The second term $\sum _{v=1}^V R_vE_v$ represents the income from earliness if the finishing time of vessel is earlier than the due time. The last term $\sum _{v=1}^V U_v|P_v-\hat{P}_v|$ represents the cost of the vessel being moored at an undesired berthing position, i.e. away from its preferred berthing position. Constraints (2) determine if a vessel is early or late depending on the difference between its due time and its finishing time. Constraints (3–7) represent the conditions for berth allocation. Constraints (3) are such that if $\delta _{v_i v_j} =1$ then the finishing time of vessel *i* is less than or equal to the berthing time of vessel *j*; 0 otherwise. Figures 2 and 3 illustrate how the value of $\delta _{v_i v_j}$ is computed. In Fig. 2, $\delta _{v_i v_j}$ takes value 1 as $F_{v_i}\le T_{v_j}$, whereas its value in Fig. 3 is 0 since $F_{v_j}> T_{v_i}$.

**Fig. 4:**
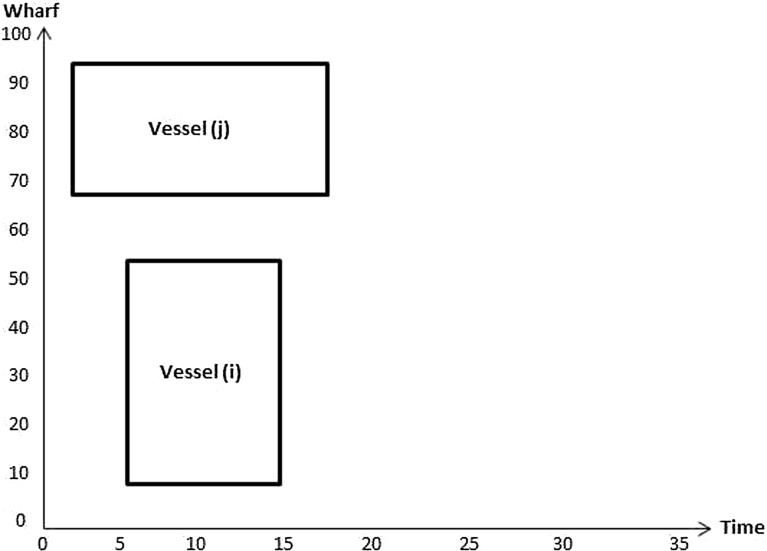
Illustration of no overlap in location between vessels *i* and *j*

**Fig. 5:**
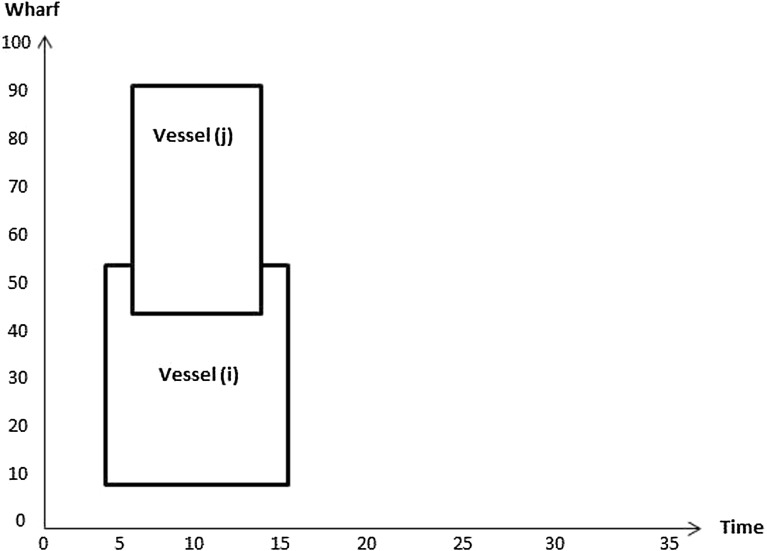
Illustration of overlap in location between vessels *i* and *j*

Constraints (4) are such that if $\sigma _{v_i v_j} =1$ then the berthing position of vessel *i* plus the length of vessel *i* is less than or equal to the berthing position of vessel *j*; 0 otherwise. Figures 4 and 5 illustrate how the value of $\sigma _{v_i v_j}$ is computed. In Fig. 4 the value of $\sigma _{v_i v_j}$ is 1 since $P_{v_i}+L_{v_i}\le P_{v_j}$. In the Fig. 5 $\sigma _{v_j v_i}$ equals 0 as $P_{v_j}+L_{v_j}\ge P_{v_i}$.

Constraints (5) ensure that the overlaps among vessels do not exist in the two dimensional space (time and location) depending on the value of $\delta _{v_i v_j}$ and $\sigma _{v_i v_j}$. Constraints (6) guarantee that vessels cannot moor before their arrivals. Constraints (7) imply that the berthing position plus the length of the vessel cannot exceed the range of the wharf.

Constraints (8) through (11) represent the main conditions for quay crane scheduling. Constraints (8) and (9) select the first and the last ships for each quay crane, respectively. Constraints (10) guarantee that ships are processed in a well-defined sequence. Constraints (11) guarantee that each vessel is handled by at least one quay crane, assuming that every docked ship comes with work.

Constraints (12) through (14) determine the starting time of quay cranes. Constraints (12) define the starting time of the earliest vessel that is to be done by the *q*th quay crane which should be after the ready time of this quay crane. Note that vessel $v_o$ is a dummy vessel. Constraints (13) state that the starting time of the *q*th quay crane on vessel *v* is no earlier than its berthing time if the *q*th quay crane is assigned to it. Constraints (14) ensure that the starting time of the *q*th quay crane on vessel $v_j$ should be no earlier than the finishing time of the preceding vessel $v_i$.

Constraints (15) ensure that if a quay crane is allocated to a vessel, then it will start its processing with a single task on that vessel. Constraints (16) ensure that if a quay crane is allocated to a vessel, then it will finish its processing again on a single task on that vessel. Constraints (17) show a flow balance ensuring that tasks are performed in a well-defined sequence on every single vessel. Constraints (18) ensure that every task on every vessel must be completed by exactly one quay crane. Constraints (19) ensure that if a quay crane is not assigned to a vessel, the tasks on this vessel will not be performed by this quay crane. Constraints (20) determine the completion time of each task. Constraints (21) define the quay crane operation starting time. The completion time of each quay crane is computed by constraints (22). Constraints (23) specify the finishing time of each vessel.

When required, Constraints (24) force task *i* to be completed before task *j* for all the tasks which are in the set $\Phi$. Constraints (25) are such that $Z_{ij}^{v}=1$ when the operation of task *j* on vessel *v* starts after the operation for task *i* is completed; and 0 otherwise. Constraints (26) ensure that the pair of tasks that are members of the set $\Psi$ are not handled simultaneously.

Constraints (27) are interference avoidance constraints between quay cranes. Suppose that tasks *i* and *j* are performed simultaneously and $l_i<l_j$, this means that $Z_{ij}^{v}+Z_{ji}^{v}=0$. Note that both quay cranes and tasks are sorted in an increasing order of their relative location in the direction of increasing ship-bay numbers. Suppose furthermore that, for $q_1 <q_2$, quay crane $q_1$ performs tasks *j* and quay crane $q_2$ performs task *i*. Then, interference between quay cranes $q_1$ and $q_2$ results. However, in such a case, $\sum _{\theta =1}^{q_1}\sum _{\kappa =0}^{Nv}X_{\kappa j}^{\theta v}-\sum _{\theta =1}^{q_1}\sum _{\kappa =0}^{Nv}X_{\kappa i}^{\theta v}=1$, which cannot be allowed because of Constraints (27), and so we have $Z_{ij}^v+Z_{ji}^v=0$.

**Fig. 6:**
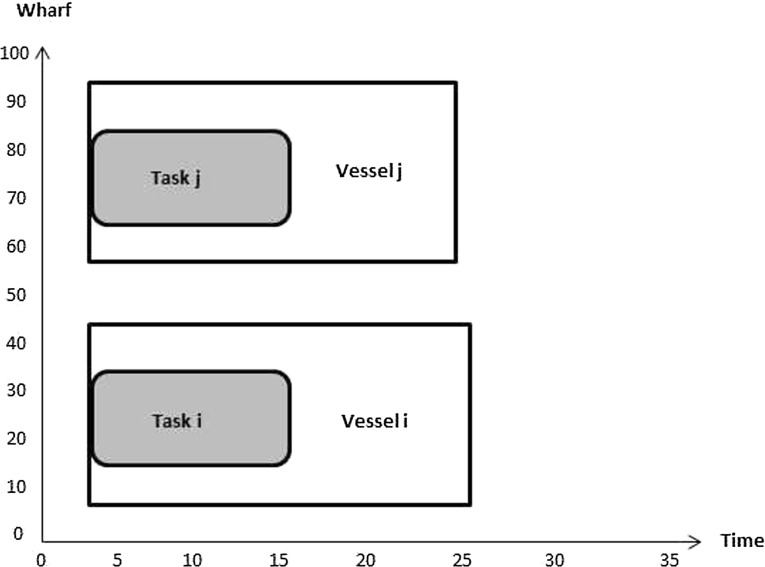
No location overlap between two vessels

**Fig. 7:**
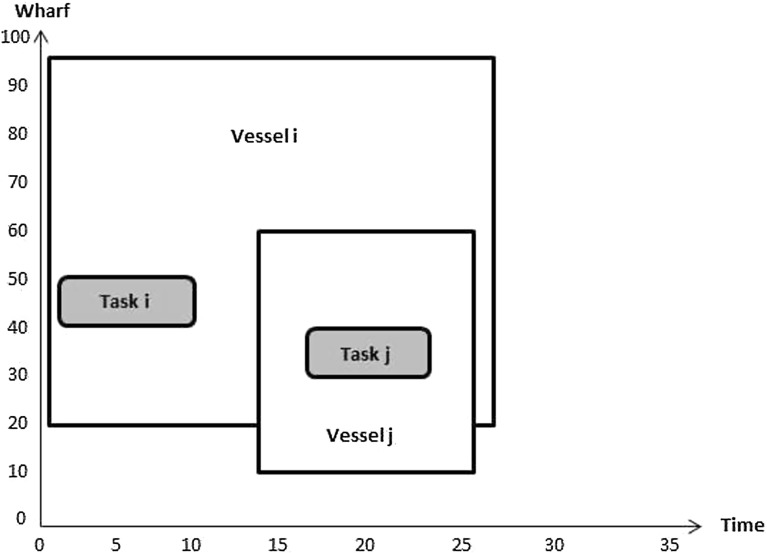
Location overlap between two vessels

Constraints (28) through (30), which are introduced here for the first time, enforce interference avoidance between different tasks on different vessels; they enable quay cranes to move freely between vessels. Constraints (28) are such that $\alpha _{b_ib_j}^{v_i v_j}$ takes value 1 if the sum of the berthing position of vessel $v_i$ and the location of task $b_i$ on that vessel is less than or equal to the berthing position of vessel $v_j$ plus the location of task $b_j$ on that vessel; 0 otherwise. Figures 6 and 7 illustrate how the value of $\alpha _{b_ib_j}^{v_i v_j}$ is computed.

The value of $\alpha _{b_ib_j}^{v_i v_j}$ in Fig. 6 is 1 because $P_{v_i}+l_{b_i}^v\le P_{v_j}+l_{b_j}^v$. Its value in Fig. 7 is 0 since $P_{v_i}+l_{b_i}^v> P_{v_j}+l_{b_j}^v$. This means that there is position overlap between these two tasks on the two vessels.

**Fig. 8:**
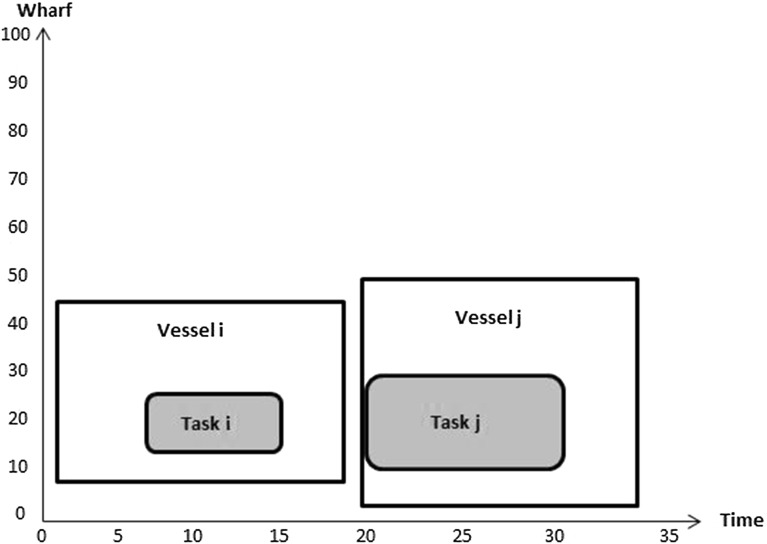
No time overlap between two vessels

**Fig. 9:**
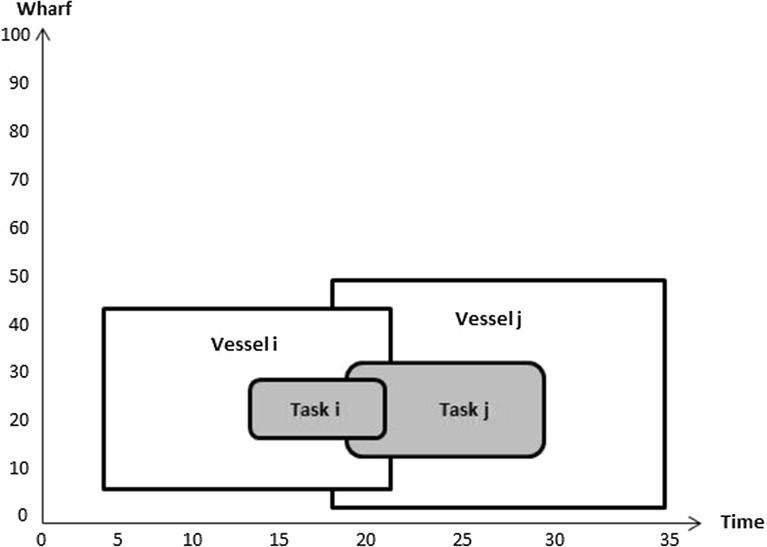
Time overlap between two vessels

Constraints (29) are such that $\beta _{b_ib_j}^{v_i v_j}=1$ if the finishing time of task $b_i$ on vessel $v_i$ plus the processing time of task $b_j$ on vessel $v_j$ is less than or equal the finishing time of task $b_j$ on vessel $v_j$; 0 otherwise. Figures 8 and 9 illustrate how the value of $\beta _{b_ib_j}^{v_i v_j}$ is computed. In Fig. 8 it is 1 because $D_{b_i}^{v_i}+p_{b_j}^{v_j}\le D_{b_j}^{v_j}$. However, the value of $\beta _{b_jb_i}^{v_j v_i}$ in the same figure is 0 since $D_{b_j}^{v_j}+p_{b_i}^{v_i}> D_{b_i}^{v_i}$.

The value of $\beta _{b_ib_j}^{v_i v_j}$ in Fig. 9 equals 0 because $D_{b_i}^{v_i}+p_{b_j}^{v_j}> D_{b_j}^{v_j}$ and the value of $\beta _{b_jb_i}^{v_j v_i}$ in the same figure equals 0 because $D_{b_j}^{v_j}+p_{b_i}^{v_i}> D_{b_i}^{v_i}$. This means that there is overlap in time between these two tasks on these two vessels.

Constraints (30) prevent the interference between quay cranes and vessels based on the values of $\beta _{b_ib_j}^{v_i v_j}$ and $\alpha _{b_ib_j}^{v_i v_j}$, respectively.

### Numerical examples

To illustrate the value of allowing quay cranes to move, in terms of the quality of plans, consider the situation in which two quay cranes are available to handle two vessels with data as given in Table 1. Each vessel has two tasks to carry out.

**Table 1:** Input data of Example 1

Ready (crane)	0	0
Initial location (crane)	11	14
Processing time of tasks, vessel 1	85	27
Processing time of tasks, vessel 2	18	33
Location task, vessel 1	1	3
Location task, vessel 2	1	4
Expected departure time of vessel	85	33
Berthing time	0	0
Berthing position	10	15
Tardiness cost (per unit time)	5	1
Earliness income (per unit time)	1	1

**Fig. 10:**
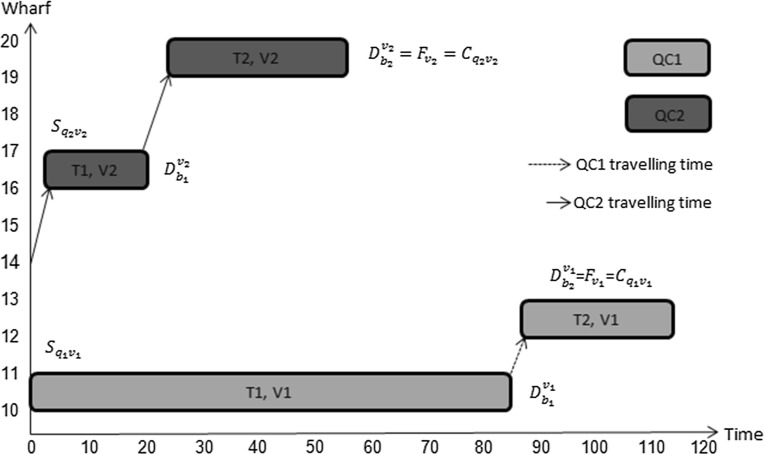
Illustration of fixed allocation of QC’s

**Fig. 11:**
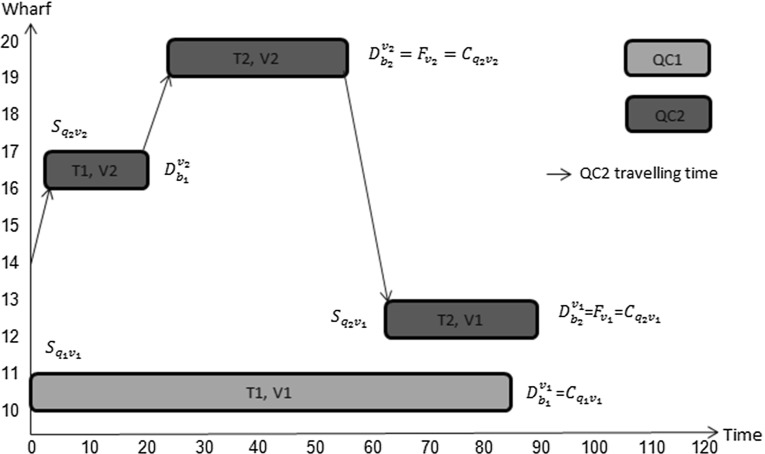
Illustration of flexible allocation of QC’s

When a fixed number of quay cranes is allocated to every vessel during the whole processing period, the optimal working plan is described in Fig. 10. Even though quay crane 2 finished its work on vessel 2 at $(2+18+3+33)=56$ time units, it is not allowed to move away from vessel 2. This wastes its effective working time which results into a sub-optimal solution. In contrast, in our model, a variable number of quay cranes is used during the processing period, which allows quay crane 2 to move from vessel 2 to perform other tasks as shown in Fig. 11. As a result, the finishing time for vessel 1 is $56+6+27=89$ time units which is earlier than in the previous plan where it is $85+2+27=114$ time units, due to making the best use of both quay cranes. Note that we only allow the quay crane to move after it has finished its work on vessel 2.

Our model also allows quay cranes to share tasks on the same vessel to which they are allocated. Consider the input data for two vessels arriving at a container terminal as given in Table 2.

**Table 2:** Input data of Example 2

Ready (crane)	0	0
Initial location (crane)	22	24
Processing time of tasks, vessel 1	28	22
Processing time of tasks, vessel 2	39	34
Location task, vessel 1	1	2
Location task, vessel 2	1	2
Expected departure time of vessel	28	39
Berthing time	0	0
Berthing position	20	25
Tardiness cost (per unit time)	1	1
Earliness income (per unit time)	1	1

**Fig. 12:**
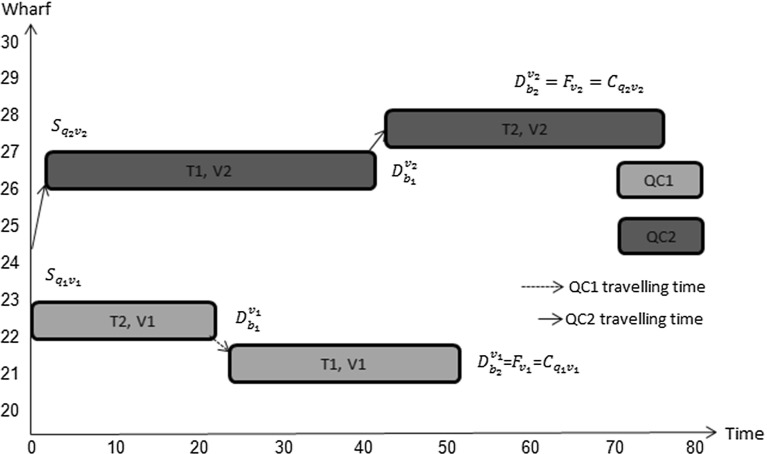
Illustration of fixed allocation of QC’s

**Fig. 13:**
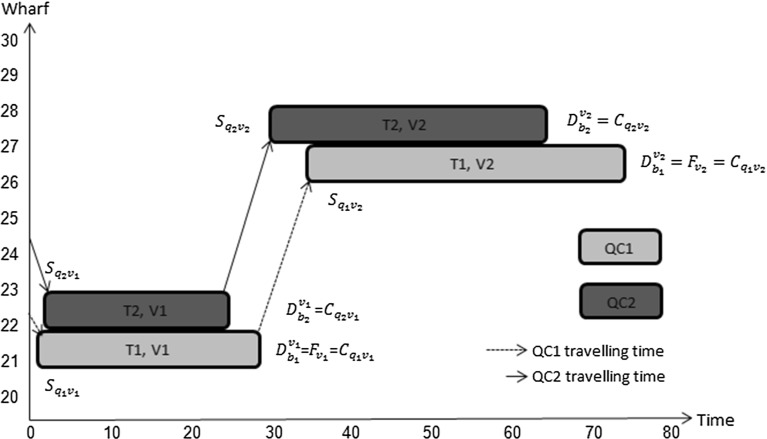
Illustration of flexible allocation of QC’s

In Fig. 12, the finishing time for vessel 1 is equal to $28+1+22=51$ time units and the finishing time for vessel 2 is equal to $2+39+1+34=76$ time units. Since we allow quay cranes to move between vessels if no interference constraints are violated, the solution to our model is the finishing time of task 1 on vessel 1 which is $1+28=29$ time units whereas the second task on the same vessel will be performed by the second quay crane with finishing time equal to $2+22=24$ time units, and the finishing time of task 1 on vessel 2 which is $29+5+39=73$ time units and the finishing time of task 2 on vessel 2 which is $24+5+34=63$ time units. As a result the finishing time of vessel 1 will be at 29 time units and the finishing time of vessel 2 will be at 73 time units, as can be seen in Fig. 13.

BAP and QCSP have been shown to be NP-hard in Ak and Erera (2006), Lee et al. (2008) and Garey and Johnson (1979). Combining them with QCAP does not reduce the complexity of the joint problem since scheduling has still to be done. If anything, the increase in the size of the combined model due to the additional variables, some of which binary, and constraints, makes the problem no less difficult. Therefore, large scale instances cannot be solved efficiently to optimality with exact approaches. Hence the need for an approximate approach. Here, we choose the genetic algorithm. GA is a well established meta-heuristic, among many others, (Salhi 2017), which has been shown to be very effective on combinatorial optimisation problems of the same type as BACASP, such as QCASP, (Alsoufi et al. 2017; Vazquez-Rodríguez and Salhi 2006a, b). Hence the choice. Given the novelty of the BACASP model that we have put forward, an innovative implementation of GA is necessary to solve it. This implementation is explained below.

## Application of the genetic algorithm to BACASP

Once an instance of the model is defined and the data is available, submitting it to CPLEX is straightforward. However, to apply GA requires that we define a representation of the problem solutions as well as other algorithmic components necessary for its implementation. GA, proposed by Holland (1975), is a stochastic and adaptive heuristic method based on evolution through natural selection ideas due to Darwin and others. It is a population based approach, i.e. it searches for solutions by maintaining a population of solutions that are then updated from generation to generation using a number of genetic operators such as Crossover, Mutation, and Reproduction. Over successive generations the population evolves towards an optimal or near-optimal solution. The processes involved in a GA are outlined below (Kaveshgar et al. 2012).

1. Generate an initial population: solutions (chromosomes) are created randomly to form this population.
2. Evaluate the fitness of each individual: choice of parents of new individuals for the new generations is biased toward individuals with good fitness values.
3. Create children: generate new individuals using genetic operators such as crossover, mutation and reproduction.
4. Generate new population: replace the worst individuals in the population with new and better ones.
5. Stopping: the process is repeated until stopping criteria are met; these may include a specified maximum number of generations or a time limit, good enough fitness value etc...

### Solution representation: chromosome

GA starts with a randomly generated population of solutions. Each solution is called a chromosome and consists of a sequence of genes. A solution or chromosome for BACASP here is a strand of genes made of three parts. The first part represents berthing times ($T_v$) of vessels. The second part represents berthing positions ($P_v$). Berthing times and positions are generated randomly but within the feasible solution set defined by the constraints (6), (7) and (32) for each solution $((T_v), ( P_v))$. The third part here represents a sequence of holds (tasks) for all docked vessels. The value of a gene is randomly picked from the index set of all holds; each gene is unique. Each chromosome consists of $v\times b$ genes, where *v* represents the number of vessels and *b* the number of tasks (bays) on each vessel. The chromosomes are character rather than binary strings.

A simple chromosome for the case of three vessels, each with three tasks, is illustrated in Fig. 10. Genes 1–3 represent berthing times. Genes 4–6 represent berthing positions, and genes 7–12 represent the sequence of tasks to be carried out on the three vessels (Table 3).

**Table 3:** Chromosome representation

Chromosome	42	37	65	213	185	370	1	4	3	6	5	2
$T_i \& P_i$	$T_1$	$T_2$	$T_3$	$P_1$	$P_2$	$P_3$	Sequence of tasks					

Based on the sequence of tasks of all vessels represented by the chromosome, a quay crane schedule can be constructed using the following steps that are an extension of the procedure proposed by Lee et al. (2008) and used for each vessel separately. Here, however, we assume that the berth allocation plan (berthing time and berthing position of each vessel) and the initial position of each quay crane at the beginning of scheduling, are known.

### Quay crane scheduling procedure (Lee et al. 2008)

- *Step 1* Based on the current position of each quay crane, determine which quay cranes can handle the first unassigned task in the chromosome without interference with other quay cranes. If only one quay crane is available, this task is assigned to this quay crane and it is deleted from the chromosome; the position and the completion time of the assigned quay crane are updated. The completion time of task *i* is also computed. If two quay cranes are available, go to Step 2.
- *Step 2* Compare the completion times of the two available quay cranes, and assign this task to the quay crane with earlier completion time. This task is then deleted from the chromosome, and both the position and the completion time of the assigned quay crane are updated. The completion time of task *i* is computed. If their completion times are equal, go to Step 3.
- *Step 3* Compare the distances between this task and the two available quay cranes, respectively, and assign the task to the quay crane with the shorter distance. This task is deleted from the chromosome, and both the position and the completion time of the assigned quay crane are updated. The completion time of task *i* is also computed. If the distances are equal, go to Step 4.
- *Step 4* Assign this task to the quay crane with the smaller order number. Then, delete this task from the chromosome, and update both the position and the completion time of the assigned quay crane. Compute the completion time of task *i*.
- *Step 5* Steps 1–4 are repeated until all the tasks in the chromosome are assigned.

### Solution validation

To validate chromosomes/solutions, four important situations must be considered. The first one is that each generated random solution is checked against constraints (5) to see that there is no overlapping of ships in time and location. A solution that satisfies these constraints is accepted. Otherwise it is accepted after addition of a penalty term to its objective function value *Z*. The objective function value and the penalty term, when used, form the fitness of that solution. The penalty term in the fitness function gradually removes infeasible solutions from the next generations. The second situation is the precedence relationship between tasks. For instance, some of the bays of a given vessel need to be unloaded and loaded. The discharging of containers from a bay must precede the loading of the bay. For this reason the generated chromosome should be checked to see if it satisfies this condition, i.e. constraints (24). The third situation is the non-simultaneity of some of the tasks, i.e. constraints (26) must also be satisfied. Finally, the interference between quay cranes is avoided by introducing non-interference constraints which consider both the potential interference of tasks on the same vessel (constraints 27) and those on different vessels (constraints 30). If either of constraints (5), (24), (26), (27) or (30) are violated or all are violated, the generated chromosomes are discarded by adding a high penalty to their fitness values.

### Evaluation of fitness

The objective of BACASP is to minimise the cost of using quay cranes and the tardiness of vessels. The solution consists in finding the optimum berthing time and position for each vessel arriving at a container terminal. The completion time of a quay crane is computed by summing up the processing times of all the tasks that have been performed by this quay crane plus the travel time it takes to move from one hold to another. The tardiness of each vessel can be computed by subtracting its finishing time from its expected departure time. The finishing time represents the maximum processing time of the vessel required by the quay cranes assigned to it. The objective function used by the GA in MATLAB is the same objective function as that of the mathematical model. Thus, the fitness value of a chromosome is calculated as

$$\begin{aligned} {\textit{Fitness}}\, ({\textit{chromosome}}) = \frac{1}{\sum _{v=1}^V W_vA_v-\sum _{v=1}^V R_vE_v+\sum _{v=1}^V U_v|P_v-\hat{P}_v|}. \end{aligned}$$

### Generating the next population

From an initial population of solutions and their fitness values a new population is generated using the genetic operators of crossover, mutation, and reproduction. The way in which individuals are selected for participation in generating the new population and the way genetic operators are applied, are given below.

#### Selection process

To give priority to the best chromosomes to pass their genes into the next generation, the fitness proportionate selection approach is implemented using a roulette wheel. High fitness individuals/chromosomes/solutions have high probability of being selected to contribute to the next population.

#### Crossover operator 

Also called the recombination operator, it is applied to the chromosomes of a randomly selected couple of individuals (parents). The recombining of their genes results in a couple of new chromosomes (children). The way the recombination is implemented is as follows. If the specified genes are related to the variables $T_i$ and $P_i$, two random integers are chosen from interval [0, 2|*V*|], where 2|*V*| is the length of a chromosome. These two integers point to two shear points in the chromosome, one low and one high. The genes to the left of the low shear point and those to the right of the high shear point of the first parent are copied into the chromosome of the first child. In the same way, using the same shear points, the chromosome of the second parent contributes to the chromosome of the second child. The genes between the shear points are generated using a decimal random number $\lambda \in$[0,1] as follows,

$$\begin{aligned} Ch1= & {} [\lambda \times Par1+ (1-\lambda ) \times Par2], \text{ and } \\ Ch2= & {} [\lambda \times Par2+ (1-\lambda ) \times Par1] \end{aligned}$$

where *Par*1 and *Par*2 are the middle sections of the first parent and the second parent, respectively, and *Ch*1 and *Ch*2 are the corresponding middle sections of the first and second child, respectively. It is clear that if *Par*1 and *Par*2 belong to a convex possibility set related to constraints (6), (7), and (32), *Ch*1 and *Ch*2 will be in this set too. This is because if *x* is the linear combination of two integer numbers *y* and $z(\ge y)$, and $y \le | x |\le z-1$, and then $y \le | x |+1\le z$. Equations (6), (7), and (32) are then checked for feasibility to confirm the new chromosomes.

If the specified genes are related to the variables $X_i$, the ‘Order Crossover’ of Gen and Cheng (1997) is used. Order crossover is permutation-based. It works as follows. A subsequence of consecutive alleles from Parent 1 is selected and used to partially make the offspring; the remaining alleles to complete the creation of the offspring are chosen from Parent 2 avoiding any repetitions. The same procedure is then applied starting from Parent 2 to make the second offspring. For illustration purposes, please see Tables 4 and 5.

**Table 4:** Offspring1

Parent1	25	9	40	**12**	**76**	**52**	**39**	64	2	8	**4**	**9**	**12**	7	3	10	6	1	5	11
Offspring1	25	9	40	**21**	**69**	**46**	**31**	64	1	7	**4**	**9**	**12**	11	3	5	10	2	8	6
Parent2	50	25	33	**60**	**43**	**22**	**2**	39	4	12	**9**	**1**	**7**	11	3	5	10	2	8	6

**Table 5:** Offspring2

Parent1	25	9	40	**12**	**76**	**52**	**39**	64	2	8	**4**	**9**	**12**	7	3	10	6	1	5	11
Offspring2	50	25	33	**50**	**49**	**28**	**9**	39	2	8	**9**	**1**	**7**	4	12	3	10	6	5	11
Parent2	50	25	33	**60**	**43**	**22**	**2**	39	4	12	**9**	**1**	**7**	11	3	5	10	2	8	6

#### Mutation operator

Mutation is another operator of GA which helps prevent premature convergence and promote diversity in the population. Equivalently, it helps avoid getting trapped in local solutions. Its implementation consists in changing (mutating) a randomly selected gene of a given chromosome in the current population. The mutation presented in this paper is that, if the specified gene is related to the variables $$T_i$$, a random integer in $$[A_i , LN] $$, where *LN* is a large number, is chosen, and if it is related to the variables $$P_i$$, a random integer in the interval $$[0 , W - L_i]$$, is chosen. This number replaces the pervious value of that gene. In this case, every new chromosome will satisfy constraints (6), (7) and (32). If it is related to the sequence of tasks, two genes from the chromosome are randomly selected and then swapped. Table 6 illustrates the mutation operator.

**Table 6:** Mutation operator

**22**	45	73	10	**34**	66	**1**	5	3	2	4	8	7	**9**	6
**14**	45	73	10	**15**	66	**9**	5	3	2	4	8	7	**1**	6
Berthing times			Berthing positions			Sequence of tasks								

#### Reporoduction

The reproduction operator consists in copying a proportion of the current individuals as they are into the new population.

### Stopping criterion

GA stops when the maximum number of generations is reached.

## Computational experiments

Twenty instances of the above mathematical model of BACASP with different numbers of vessels, tasks, and quay cranes have been solved using CPLEX and our implementation of GA. Their statistics are recorded in Tables 7 and 8. All instances have randomly generated processing times of tasks for each hold from the uniform distribution *U*(10,50).

CPLEX solved problems 1–10 which are relatively small in size. Six problems were solved in acceptable times, but problems 6, 7, 9 and 10 required 24, 22, 24 and 60 h, respectively. These times are hardly acceptable in the context of container terminal operations. The rest of the problems, 11–20, which are real world instances, could not be solved with CPLEX in acceptable times (>100 h of CPU time). In some cases they could not be solved at all due to the limitations of the computing platform used. Therefore, we terminated the CPLEX execution on these instances after 3 h.

GA, coded in Matlab, managed to solve all 20 instances. For the small size problems of Table 7, the GA parameters of population size, rate of crossover, rate of mutation, and the maximum number of generations are set as 200, 0.5, 0.4, and 500, respectively. In the case of the large size instances of Table 8, they are 500, 0.5, 0.4, and 1000, respectively. Note that this means that 10% of the new population is generated through reproduction or simple copying.

All experiments have been performed on a PC with Intel Core i5 and 3.20 GHz CPU with 8 GB RAM running Windows 7 Operating System. Comprehensive experimental results are presented in Tables 7 and 8. The average objective function values of the solutions returned by GA and their standard deviations are recorded in columns 11 and 12 of Tables 7 and 8. CPLEX managed to solve only few of the small size instances. GA, however, found the optimal or near solutions for all instances in reasonable CPU times (see columns 13 of Tables 7, 8). The average gap between CPLEX and GA returned objective values is about 0.3%.

**Table 7:** Computational results for small scale instances

No.	Problem statistics			Problem size			CPLEX		GA				$\mathrm{Gap}\,(\%)^\mathrm{b}$
	Vessels	Tasks	Q. cranes	Constraints	Dec.vars	Int.vars	Obj. Val	CPU (hh:mm:ss)	Best obj.val	Mean	SD	CPU (s)	
1	2	2	2	120	90	66	$67^\mathrm{a}$	00:00:01	67	67	0.0	37	0.0
2	2	4	4	616	322	288	$58^\mathrm{a}$	00:02:21	58	58	0.0	41	0.0
3	2	4	5	822	374	337	$470^\mathrm{a}$	00:03:18	470	478.5	3.6	40	0.0
4	4	2	5	1038	444	373	$1250^\mathrm{a}$	00:05:33	1251	1251	0.0	42	0.08
5	3	3	5	1123	453	398	$795^\mathrm{a}$	00:41:25	805	805	0.0	41	1.25
6	3	4	4	1325	597	544	275	24:57:28	258	269	8.8	43	–
7	4	3	4	1412	624	556	$1615^\mathrm{a}$	22:23:14	1636	1636	0.0	43	1.3
8	4	2	8	2136	612	520	$1165^\mathrm{a}$	00:01:12	1166	1171.2	4.3	40	0.08
9	3	6	4	2741	1161	1102	771	24:20:17	755	777	14.1	49	–
10	3	5	8	5920	1296	1220	815	60:12:54	780	819.5	23.4	48	–

$${}^\mathrm{a}$$ Optimal solution

$${}^\mathrm{b}$$ $${\textit{Gap}}=\frac{{\textit{GA Obj.Fun.-CPLEX Obj.Fun.}}}{{\textit{CPLEX Obj.Fun.}}} \times 100$$

In the instances considered, the number of constraints, the number of decision variables, and CPLEX computational time grow exponentially with the increase in the number of vessels. However, the CPU time required by GA does not grow that fast with the increase in the problem size. CPLEX did not solve some of the larger size instances, shown in Table 8. GA, on the other hand, finds the optimal or near optimal solutions for all the instances in a reasonable CPU times (see column 13 of Table 8).

**Table 8:** Computational results for large scale instances of BACASP

No.	Problem statistics			Problem size			CPLEX		GA			
	Vessels	Tasks	Q.Cranes	Constraints	Dec.vars	Int.vars	Obj. Val	CPU (hh:mm:ss)	Best obj.val	Mean	SD	CPU (s)
11	4	8	6	16,212	3764	3662	476	03:00:00	263	300.6	19.4	280
12	5	5	5	7620	2175	2070	1220	03:00:00	1202	1237.4	40.4	244
13	4	10	8	42,072	6628	6504	698	03:00:00	358	453.05	38.60	363
14	4	10	10	63,876	7572	7434	1803	03:00:00	157	180	12.2	342
15	5	8	10	66,535	7005	6840	*	03:00:00	138	165.4	13.3	337
16	6	6	12	79,188	6222	6012	259	03:00:00	26	35.05	7.57	296
17	6	8	10	97,964	9246	9046	*	03:00:00	196	226.9	22.87	356
18	4	12	12	129,872	11,956	11,796	*	03:00:00	107	131.3	11.3	382
19	6	10	12	217,488	15,342	15,108	*	03:00:00	340	378.2	18.2	436
20	5	16	12	374,689	28,455	28,232	*	03:00:00	529	572	22.2	535

* No output is generated

## Conclusion

Determining the optimal berthing time and the best berthing position for vessels arriving at container terminals is essential for the efficient running of the these terminals. Assigning the appropriate number of quay cranes to each vessel and finding the optimum sequence in which to perform tasks are important as well. However, doing this by considering each problem individually is likely to result in suboptimal solutions. This paper describes BACASP an integrated mathematical model of these three problems namely BAP, QCAP, and QCSP, which is of the Mixed Integer Programming type. It also puts forward an evolutionary solution approach to it. Unlike existing models, BACASP allows quay cranes to move between two holds of the same ship and between two holds on different vessels. It also has other features which are not commonly represented in existing models such as interference avoidance between cranes.

Travel times of a quay crane between two holds on the same ship and between two holds on different vessels have been considered. Quay crane interference avoidance is explicitly represented as constraints 27 when the quay cranes operate on the same vessel and as constraints 30 when quay cranes are on different vessels. Another advantage of this model is that it allows a quay crane that has become idle (finished its work) to move from its current vessel to another even if overall the current vessel to which it has been allocated is still being processed. In practice, quay cranes do not always start their work at the same time. For this reason a quay crane ready time has been considered in the mathematical model. The initial position for each quay crane is taken into account in order to compute the exact time at which a quay crane is travelling. Precedence and simultaneity constraints are taken into consideration as well.

Extensive experimentation has been carried out. CPLEX 12.6 has been used to find the optimal solutions of relatively small instances of BACASP. It cannot cope with larger instances of the problem which are of practical size. GA, however, coped well with all problems. It required almost the same CPU time for all problems of small size and CPU times of the same magnitude for the larger instances. Moreover, on most of the 15 instances that CPLEX managed to solve, GA also found the optimum. Overall the average discrepancy between the objective function values of the solutions found by both algorithms is 0.3. This shows that GA, while substantially more efficient than CPLEX, it is also quite robust on most instances considered as this average discrepancy shows.

The combined model presented here is obviously the way forward as it is more likely to provide better solutions than those found by solving the seaside operations problems individually. It is also a substantial improvement on combined variants which do not allow quay cranes to move between vessels.

As said earlier, piecemeal solution of container port problems by isolating them and solving them individually will almost certainly lead to suboptimal solutions. This goes for all container port problems, including those on the landside having to do with the storage area for instance. Integrating seaside and landside problems is therefore the way forward given the availability of powerful heuristics and hardware. Another aspect of real world applications such as described here, is uncertainty. In this paper we have assumed that all data is known in advance and fixed, i.e. we have built a deterministic model. In the real world, fluctuations in arrival times, variation in the throughput of quay cranes, maintenance requirements of facilities create a lot of uncertainty. A worthwhile endeavour would be to consider a stochastic BACASP and develop appropriate solution approaches.
